# Detection of triple vessel coronary artery disease by visual and quantitative first pass CMR myocardial perfusion imaging in the CE-MARC study

**DOI:** 10.1186/1532-429X-13-S1-O29

**Published:** 2011-02-02

**Authors:** Neil Maredia, Sven Plein, John F Younger, Julia M Brown, Jane Nixon, Colin C Everett, John P Ridgway, Aleksandra Radjenovic, Catherine J Dickinson, John Biglands, Abdulghani Larghat, Stephen G Ball, John P Greenwood

**Affiliations:** 1 University of Leeds, Leeds, UK; 2 Royal Brisbane and Women's Hospital, Brisbane, Australia; 3 Leeds General Infirmary, Leeds, UK

## Objective

To compare single photon emission computed tomography (SPECT) and cardiac magnetic resonance (CMR) myocardial perfusion imaging for the detection of myocardial ischaemia in patients with triple vessel coronary artery disease (3VD) from a prospectively acquired cohort of patients with suspected coronary heart disease (CE-MARC study).

## Background

3VD is associated with an adverse prognosis, which may be ameliorated by coronary artery revascularisation. Some patients with 3VD may not be identified by visual myocardial perfusion analysis due to the phenomenon of balanced ischaemia. CMR first-pass perfusion with its superior spatial resolution may be more effective than SPECT in identifying 3VD.

## Methods

Thirty-nine patients with 3VD at X-ray coronary angiography and 39 matched patients with no significant coronary disease were identified from the CE-MARC study population [1]. Patients were matched by age, gender, hypertension and diabetes. CMR adenosine stress perfusion imaging was undertaken using a saturation-recovery gradient echo pulse sequence producing three image slices per R-R interval. Visual and Fermi deconvolution-derived CMR myocardial perfusion reserve (MPR) analyses were performed. Gated SPECT imaging was performed and interpreted by an experienced observer blinded to other test results.

## Results

**Figure 1:**
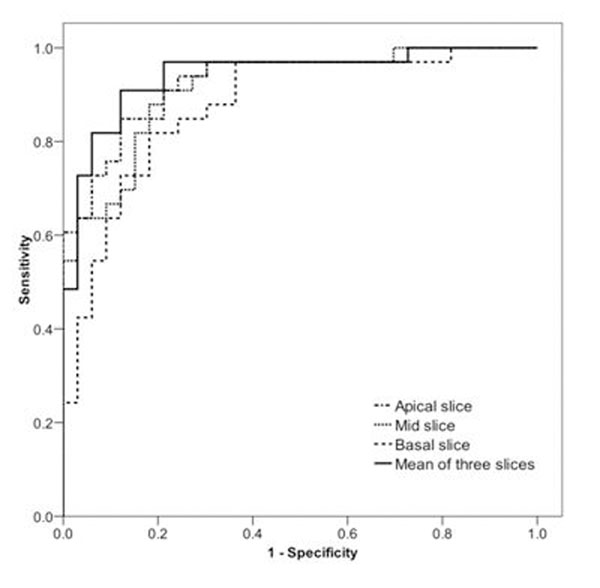
Subendocardial MPR ROC Curves

By per-patient analysis the sensitivity and specificity of visual CMR and SPECT analyses for the detection of ischaemia did not differ significantly (92% and 97% vs 82% and 91% respectively). Among the 3VD patients, 34% had hypoperfusion in all three coronary artery territories by visual CMR analysis and 18% by SPECT (p=0.14). There were significant differences in mean transmural MPR (2.16 vs 1.35; p<0.001) and subendocardial MPR (2.13 vs 1.28; p<0.001) between 3VD and normal patients respectively. Receiver operator characteristic curve analysis produced an area under curve of 0.94 for the mean MPR of all three slices and 0.92 for midventricular slice subendocardial values alone (p=NS). A mid-slice subendocardial MPR cut-off of 1.76 yielded 97% sensitivity, 70% specificity and 96% negative predictive value for the detection of ischaemia. Figure 1, Table 1.

**Table 1:** Results (a MPR cut-off <1.74; b MPR cut-off <1.76)

	Sensitivity % (95% C.I.)	Specificity % (95% C.I.)	Positive Predictive Value % (95% C.I.)	Negative Predictive Value % (95% C.I.)
SPECT	82.3 (64.8-92.6)	91.1 (75.2-97.7)	90.3 (73.1-97.5)	83.8 (67.3-93.2)
Visual CMR	92.1 (77.5-97.9)	97.3 (84.6-99.9)	97.2 (83.8-99.9)	92.5 (78.5-98.0)
Mean of 3 Slice Subendocardial MPR ^a^	97.0 (82.5-99.8)	78.8 (60.6-90.3)	82.1 (65.9-91.9)	96.3 (79.1-99.8)
Mid Slice Subendocardial MPR ^b^	97.0 (82.5-99.8)	69.7 (51.1-83.8)	76.2 (60.2-87.4)	95.8 (76.9-99.8)

## Conclusion

Patients with 3VD may be reliably differentiated from those without obstructive coronary disease by visual or quantitative CMR myocardial perfusion assessment. Visual CMR analysis performs at least as well as SPECT imaging in this regard. Fermi-derived MPR assessment may be performed efficiently using the middle slice alone without a loss of diagnostic accuracy relative to a three-slice analysis.

## References

[B1] Greenwood Trials 2009 10 62 10.1186/1745-6215-10-62 19640271 PMC3224948

